# Supplementary material for: Elevated Serotonin and NT-proBNP Levels Predict and Detect Carcinoid Heart Disease in a Large Validation Study
Source: Cancers (Basel). 2022 May 10;14(10):2361. doi: 10.3390/cancers14102361 (PMC9139809; doi:10.3390/cancers14102361)
Supplement: Supplementary file 1 [file cancers-14-02361-s001.zip › cancers-1631253-supplementary.pdf]

Supplementary Materials

Elevated Serotonin and NT-proBNP Levels Predict and Detect Carcinoid Heart Disease in a Large Validation Study

Sonja Levy [1,*,†], Aoife B. Kilgallen [2,3,†], Catharina M. Korse [4], Marish I. F. J. Oerlemans [3], Joost P. G. Sluijter [2,3,5], Linda W. van Laake [2,3], Gerlof D. Valk [6,†] and Margot E. T. Tesselaar [1,†]

[1] Department of Medical Oncology, Netherlands Cancer Institute, 1066 CX Amsterdam, The Netherlands; m.tesselaar@nki.nl
[2] Regenerative Medicine Centre Utrecht, Circulatory Health Laboratory, University Medical Centre Utrecht, 3584 CX Utrecht, The Netherlands; a.kilgallen@umcutrecht.nl (A.B.K.); j.sluijter@umcutrecht.nl (J.P.G.S.); l.w.vanlaake@umcutrecht.nl (L.W.v.L.)
[3] Division of Heart and Lungs, Department of Cardiology, Experimental Cardiology Laboratory, University Medical Centre Utrecht, 3584 CX Utrecht, The Netherlands; m.i.f.oerlemans-4@umcutrecht.nl
[4] Department of Laboratory Medicine, Netherlands Cancer Institute, 1066 CX Amsterdam, The Netherlands; t.korse@nki.nl
[5] Utrecht University, 3584 CS Utrecht, The Netherlands
[6] Department of Endocrine Oncology, University Medical Centre Utrecht, 3584 CX Utrecht, The Netherlands; g.d.valk@umcutrecht.nl
* Correspondence: so.levy@nki.nl; Tel.: +31-20-512-9111
† These authors contributed equally to this work.

Table S1. Individual CHD-scores per patient.

Patient	TV—regurgitation	TV—stenosis	TV—leaflet thickening	TV—leaflet excursion	TV—leaflet retraction	PV—regurgitation	PV—stenosis	PV—cusp thick	PV—cusp excursion	PV—cusp retraction	RA—dilation	RV—dilation	RV—impairment	MV—regurgitation	MV—stenosis	MV—leaflet thickening	MV—leaflet excursion	MV—leaflet retraction	AV—regurgitation	AV—stenosis	AV—cusp thickening	AV—excursion	AV—retraction	CHD—score
**1**	3		3	3	3	0	0					3	3	0	0	0	0	0	0	0	0	0	0	**18**
**2**	3		3		3	2	2	2				3		0	0	0	0	0	0	0	0	0	0	**18**
**3**	3	3			3									1					1					**11**
**4**	3	0	3			2	1					3	0	1						1				**14**
**5**	3	0				3		2				2	1	1		1			1					**14**
**6**	3		3			3	1					2	0	1		1			0					**14**
**7**	2	0	2			1	0	0						3					1					**9**
**8**	2		2																					**4**
**9**	3		2			1						0	0	3							1			**10**
**10**	3		3			3	3					2	0	1					1					**16**
**11**	2		2			0	0					0	0	1					1		1			**7**
**12**	3	2	3		3	1	0					1	0	1					1					**15**
**13**	3	0	1			1	0					0	0	2		1								**8**
**14**	3		1									0	0	1					1					**6**
**15**	3		1																					**4**
**16**	3											3												**6**

**17**	2		3		2		3	0	0	1					**11**
**18**	3							0	0				0	1	**4**
**19**	3		2					2	0	1			2		**10**
**20**	2	0	2		0	0							1		**5**
**21**	2		2					1	0	2	2		1	1	**11**
**22**	3		3		3			2	0	1					**12**
**23**	3				3			2	0	2			0		**10**
**24**	3														**3**
**25**	3	2			1	0		3		1					**10**
**26**	3	2	2		3	2		3	2	2			0		**19**
**27**	3														**3**
**28**	3		3		0	0		2	0	1					**9**
**29**	2		1						0	1					**4**
**30**	2			2	3			1	0	1			1		**10**
**31**	3		3	3					0	2			2		**13**
**32**	3			3	3			3		0			0		**12**
**33**	2			3	1			0	0	2			0		**8**
**34**	3			3				1	0	1			1		**9**
**35**	3		3		2	0		2							**10**
**36**	3		2	3				3	2				1		**14**
**37**	3														**3**
**38**	3			3	3			3	0	2					**14**
**39**	3		2	1	1			2	0				1		**10**
**40**	4				2			1							**5**
**41**	3		3		1		2	2	0	1					**12**
**42**	2		3		3		3	2	0	1					**14**
**43**	3		3	0				2	0	0			0		**8**
**44**	3		3	3	2			2	0	1			1		**15**
**45**	3														**3**
**46**	3				2										**5**
**47**	1				3		1	0	0	0					**5**
**48**	2							1		1			0		**4**
**49**	3		3	3	2			2	0			1	0		**14**
**50**	3		3	3	2			3	0	0	1		0		**15**
**51**	3		3	3	2			0	0	3	3	3	1		**21**
**52**	3		3	3				3	1						**13**
**53**	3		3		2			0	0						**8**
**54**	3		3	1				1	1						**9**
**55**	3		3	3	3	0		2	0	1			2		**17**
**56**	3							2	1						**6**
**57**	3		1		3	1		2	1	1			0		**12**

TV: tricuspid valve; PV: pulmonic valve; RA: right atrium; RV: right ventricle; MV: mitral valve; AV: aortic valve.

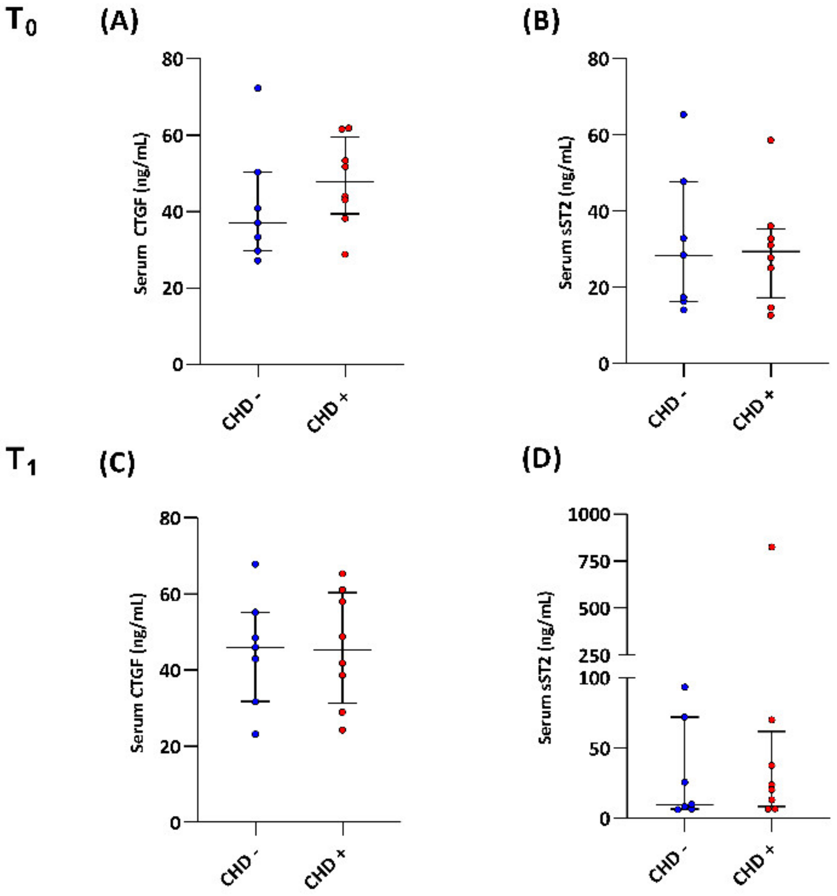

Figure S1. Serum sST2 and CTGF levels at time-points T0 and T1. Both sST2 (p = 0.867) (**A**) and CCN2/ CTGF (p = 0.232) (**B**) are not significantly different at T0. This is similar at T1 for serum sST2 (p = 0.694) (**C**) and CCN2/ CTGF (p = 0.955) (**D**).
